# Migraine Triggers: An Overview of the Pharmacology, Biochemistry, Atmospherics, and Their Effects on Neural Networks

**DOI:** 10.7759/cureus.14243

**Published:** 2021-04-01

**Authors:** Hassan Kesserwani

**Affiliations:** 1 Neurology, Flowers Medical Group, Dothan, USA

**Keywords:** food chemistry, migraine disorder, menstrual migraine, weather conditions

## Abstract

We define a migraine trigger to be an endogenous agent or agency such as the menses or an exogenous agent or agency such as red wine or a drop in barometric pressure, and their ability to reduce the threshold of a migraine attack in those predisposed to migraine. This definition excludes agents with idiosyncratic mechanisms that may trigger a migrainous (migraine-like) headache in non-migraineurs such as benign cough headaches or headaches due to altitude-sickness. We also assume as axiomatic that migraine has as its basis the activation of the trigeminovascular pathway (TVP) and the key role of serotonin and the calcitonin gene-related peptide (CGRP). The network activation of the visual/auditory association cortices and the rostrodorsal pons (locus ceruleus and raphe nucleus) are also accepted as key features of activation of the TVP. In addition, we outline the role of the superior salivatory nucleus-sphenopalatine ganglion-greater superficial petrosal nerve (SSN-SPG-GSPN) arc in migraine activation.

We also explore how olfactory afferents intermingle with trigeminal nerve collaterals in the glomeruli of the olfactory bulb thus allowing volatile molecules to activate the TVP and induce a migraine. The classification of migraine triggers is complex, as there is a wide panorama of inciting agents, including atmospheric conditions, a wide-ranging variety of foods and beverages, endogenous hormonal influences, synthetic alkaloids and dyes, and volatile molecules (odorants). We will explore the high-frequency migraine-provoking agents in each category. There are exciting and intriguing hypotheses regarding the role of atmospheric chemistry when the barometric pressure drops; the role of hot, dry desert winds and lightning discharges in the generation of cations and the turnover of serotonin in the nervous system. We will explore the effects of a drop in barometric pressure on the vestibular nuclei and the modulation of sympathetically mediated pain. The role of volatile odorants and their activation of the transient receptor potential ankyrin-1 (TRPA-1) receptor will be outlined. We will streamline the highly complex role of estrogen fluctuation in the precipitation of migraine headaches, its pharmacodynamic effects, and the role of the sexually dimorphic nucleus of the preoptic area (SDN-POA) of the hypothalamus. We will also adumbrate the protean effects of alcohol and its congeners and the role of stress and sleep disturbances in the allostatic load model of salience network-pain perception.

## Introduction and background

In one of the earliest studies of migraine triggers, 500 patients (300 females, 200 males) were interviewed, and menstruation and emotional factors were identified as the two commonest migraine triggers, followed by the glare of light and foods. Alcoholic beverages were not included in the analysis (Table 1) [1].

**Table 1:** Migraine triggers in one of the earliest large-scale studies

MIGRAINE TRIGGER	PROPORTION	PERCENTAGE	COMMENT
Menstruation	196/500	62	14% exclusively menstrual
Emotion	388/500	67	Could be an upsetting event such as an argument at home or anxiety shopping
Glare of light	293/500	47	Usually a flicker of light, as in televisIon viewing or exposure to sun
Foods	339/500	25	Fats, fried foods, chocolates, oranges, tomatoes, pineapples, and onions; alcoholic beverages were not included in this study

In one of the largest migraine trigger studies of 1027 migraineurs whose migraine was classified by the international headache society (IHS) criteria, the reported migraine triggers in descending order of frequency are listed below. The high-frequency triggers, stress and hormonal influence, mirror those found in the earliest study listed in Table 1 above. However, this study displayed a higher resolution of detail with the inclusion of weather changes, sleep disturbances, odorants, and alcohol (Table 2) [2].

**Table 2:** Migraine triggers in one of the largest migraine trigger studies

MIGRAINE TRIGGER	PERCENTAGE
Stress	79.7
Hormones	65.1
Missing a meal/hunger/fasting	57.3
Weather change	53.2
Sleep disturbance	49.8
Perfume or odor	43.7
Neck pain	38.4
Light	38.1
Alcohol	37.8
Smoke	35.7
Sleeping late	32.0
Heat	30.3
Food	26.9
Exercise	22.1
Sexual activity	5.2

In a logistic regression model using attack frequency and migraine subtype as covariates, a study of 5725 females and 1061 males analyzed the sexual dimorphism of migraine triggers by calculating odds ratios. Eleven triggers were analyzed and six (bright lights, stress, skipping a meal, sleep deprivation, high altitudes, and weather changes) were more commonly reported in women, notwithstanding menstruation. Menstruation, stress, and bright lights were the commonest triggers in women, and sleep deprivation, stress, and bright lights were the three commonest triggers in men. Overall, migraine triggers were more frequent in women and multiple triggers were also more common in women [3]. 

In a Belgian study of 217 migraineurs (176 women, 41 men), migraine triggers were more common in older women with longer disease duration. Menstruation as a trigger increased the risk of poly-triggers such as food and beverages. In descending order of frequency, the triggers were alcoholic beverages (51.6%), stress (48.8%), menses (48%), foods (47.7%), and ovulation (8.5%), again emphasizing the role of stress and hormonal influence with the inclusion of alcohol as a migraine trigger in this study [4].

In a Brazilian study of 200 migraineurs, a sleep disorder (oversleep, lack of sleep, or change in sleep pattern) was a trigger in 81% of patients and emotional stress in 64%. Menses as a trigger was reported in 53%. Dietary factors were reported in 64% of patients, with fasting being the most common trigger followed by alcohol (34%), red wine (36%), chocolate (20.50%), white wine (18%), and aspartame (14%). Of the patients, 36.5% reported an odorant (smell) as a trigger [5]. The role of a sleep disturbance is highlighted in this study, with emphasis on both oversleep and lack of sleep.

In an odorant study of 200 migraine patients, an odor-triggered migraine occurred within 30 minutes of exposure in 70% of patients. In descending order of frequency, the rates were 75.7% for perfumes, 42.1% for paints, 28.6% for gasoline, and 27.1% for bleach. Sensitivity to perfumes was correlated with sensitivity to cleaning, cooking, and beauty products. This study was powered by the fact that 200 migraineurs were compared with 200 patients with tension-type headaches, none of which experienced a headache trigger. There was also a tight correlation between osmophobia and odorant-triggered migraine [6].

In a remarkable study of 200 children, between the ages of seven and 15, from Eastern India, the highest frequency of migraine triggers was environmental, including a hot-humid climate, smoke-noise pollution, and homework/exam-related stress. Meanwhile, menses was a low-frequency trigger in girls [7].

In summary, migraine-associated triggers are common and fall into five main categories: emotional stress, menstrual-induced, sleep disturbance, food and alcoholic beverages, and weather changes.

In order to understand how migraine triggers precipitate migraine headaches, we need to understand the network theory of migraine. Knowledge of the trigeminovascular pathway (TVP) and its nodal connections with the locus ceruleus (LC) and dorsal raphe nucleus (DRN) and the visual/auditory association cortices and limbic system is imperative for understanding the influence of sleep on migraine genesis. In addition, we need to be versed with the superior salivatory nucleus-sphenopalatine ganglion-greater superficial petrosal nerve (SSN-SPG-GSPN) arc of autonomic activation. We will also adumbrate on the intermingling of the ophthalmic trigeminal afferents and the olfactory nerve endings in the glomeruli of the olfactory bulb in order to help us understand how odorants are able to trigger migraines. The potential role of the sexually dimorphic nucleus of the preoptic area (SDN/POA) in the generation of hormonally induced migraines will also be highlighted.

**Figure 1:**
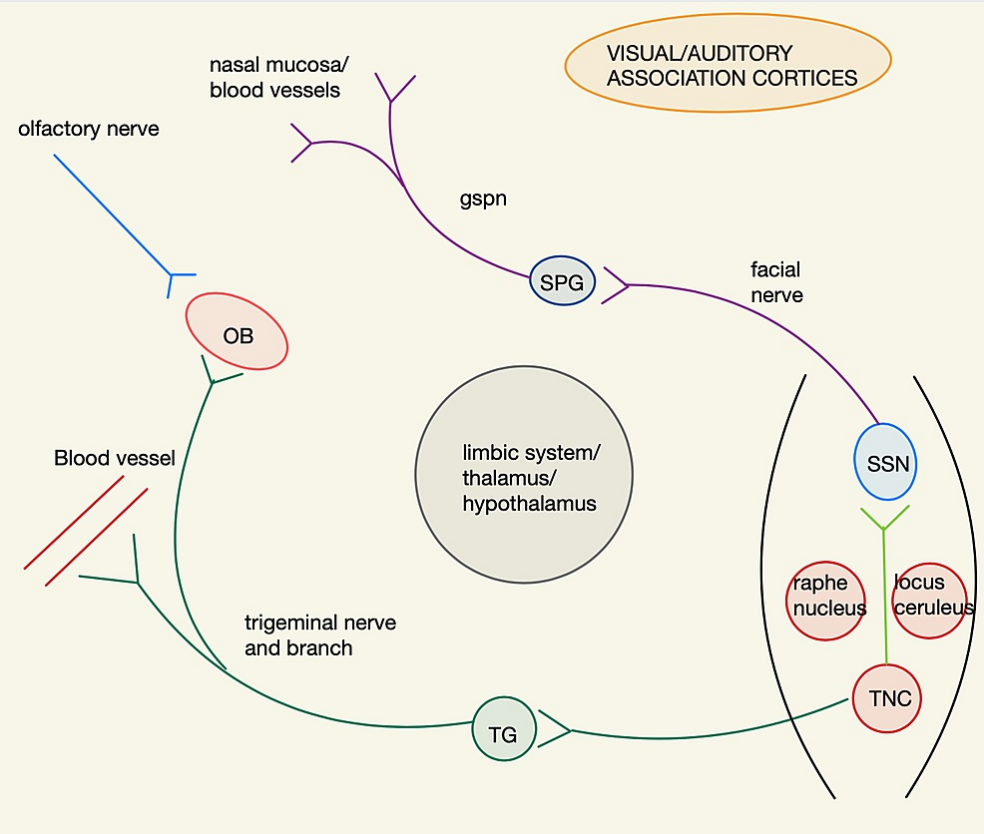
Main migraine-associated networks - simplified for clarification Olfactory bulb (OB), trigeminal ganglion (TG), trigeminal nucleus caudalis (TNC), superior salivatory nucleus (SSN), sphenopalatine ganglion (SPG), greater superficial petrosal nerve (GSPN)

Trigeminal nerve afferents activate second-order dorsal horn cells in the trigeminal nucleus caudalis (TNC), which includes the cervical C1 and C2 dorsal horn cells. Cephalad projections to the ventral-posterior-medial (VPM) thalamus and caudal projections to the spinal cord peri-aqueductal gray matter (PAG) activate pain pathways. Antidromic signaling of the trigeminovascular system (TVS) leads to the release of the inflammatory vasodilatory mediator calcitonin gene-related peptide (CGRP) and mast cell degranulation of substance P (SP) of the dural and pial vessels, respectively, leading to the activation of the small-diameter unmyelinated pain-transmitting C-fibers. These trigeminal afferents are also modulated by the serotonin, 5-hydroxytryptamine (5HT-D) receptors [8-10]. The TNC activates the superior salivatory nucleus (SSN), which by sending efferents to the sphenopalatine ganglion (SPG) leads to vasoactive intestinal peptide (VIP) release by the greater superficial petrosal nerve (GSPN), leading to lacrimation and nasal discharge [11]. These pathways (hodological networks) are illustrated in Figure 1.

In a positron-emission tomography (PET) study of nine patients with migraine, there was activation of the cingulate cortex, visual and auditory association cortices, and dorsal pons (raphe nucleus and locus ceruleus). But only dorsal pons activation persisted after sumatriptan injection. Cingulate cortex activation reflects the emotional response to pain and activation of the visual and auditory association cortices mirrors the development of photophobia and phonophobia, respectively. The raphe nucleus and locus ceruleus activation set the tone of response of the association cortices to visual and auditory stimuli [12].

In a functional magnetic resonance (fMRI) study of 15 patients with migraine without aura, there was increased connectivity between the visual/auditory cortices and anterior insula/dorsal pons. The anterior insula is a node of the salience network that sets the tone or valence of a sensory stimulus, probably accounting for the heightened sensitivity to sensory stimuli [13].

In another fMRI study of olfactory-induced migraine attacks in 20 migraine patients, there was increased blood oxygen level-dependent (BOLD) signal intensity in the insula, amygdala, and the rostral pons (locus ceruleus and raphe nucleus), highlighting the link between the olfactory system, the salience network (the insula as a node), and the trigeminovascular system (dorsal pons) [14].

## Review

Olfactory stimulation and trigeminovascular system activation: pharmacology and anatomy

The California Bay laurel tree is also known as the "headache tree." Its leaves vaporize the volatile monoterpene ketone, umbellulone, a molecule that activates the transient receptor potential ankyrin-1 (TRPA-1) peptide on nociceptive neurons of the nasal mucosa, thereby activating the trigeminovascular system directly. TRPA-1 receptors are members of the transient receptor potential (TRP) receptor family and is co-expressed with the transient receptor potential vannilin-1 (TRPV-1) peptidergic sensory nerves. TRPA-1 and TRPV-1 receptors both co-localize on nociceptive C-fibers, transduce noxious stimuli, cold temperature, and are mechanosensitive. They also respond to electrophilic agents and plant-derived volatile alkaloids. Nicotine activates and paracetamol, capsaicin, and caffeine suppress both receptors. Activation of TRPA-1 induces the release of SP and CGRP by trigeminovascular activation, explaining the generation of a migraine headache [15-17]. Olfactory stimulation also leads to increased activity of the limbic system and the dorsal pons, which induces inflammatory mediator (CGRP, SP) release and activation of nociceptors and vasodilation of meningeal vessels [14].

Odorants stimulate both the olfactory and trigeminal nerve endings in the nasal mucosa (chemosensation). The quality of a volatile molecule can elicit both an odor or a pungency (irritation). It was initially thought that the pungency of a substance may mask its odor; however, a study by Cain SW et al. showed that pungency and odor are mutually exclusive, with pungency not masking the odor of an irritant agent [18].

Chemosensation begins in the nasal mucosa. Collaterals of the ophthalmic branch of the trigeminal nerve (SP- and CGRP-positive) synapse at the glomerular layer of the olfactory bulb, where mitral cells excite each other, intimately coupling the olfactory and trigeminal pathways [19-20].

The pharmacodynamics of estrogen and migraine generation

It is well-established that migraine is three times more frequent in women than men (sexual dimorphism). The sexually dimorphic area in the hypothalamus is the pre-optic region, known as the sexually dimorphic nucleus of the pre-optic area (SDN-POA). Here, along with the paraventricular nucleus, the transition to puberty is determined by the pulsatile release of the luteinizing hormone release hormone (LHRH). Opioids inhibit the pre-ovulatory LH surge via the mu- and kappa-opioid receptors [21-22].

The relationship between migraine and estrogen is non-linear; implying that there is no straightforward proportionality. Specifically, whereas the estrogen withdrawal hypothesis (precipitation of migraine headache with a drop in estrogen levels below 40-50 pg/ml) generally holds true, this simple relationship breaks down in post-menopausal women, where hormone replacement therapy may actually increase the risk of migraine. Several days of exposure to estrogen are required prior to a drop of estrogen before a migraine is precipitated, a phenomenon known as priming [23-24].

Pharmacodynamically, estrogen increases endothelial cell nitric oxide synthetase activity and the production of nitrous oxide, a potent vasodilator. This effect heightens during the luteal phase, with a concomitant drop in serum serotonin [25]. When estrogens fall during the menses, two events occur due to membrane excitability: there is upregulation of inflammatory genes with a subsequent elaboration of cytokines and central sensitization of peptidergic neurons and increased pain [26]. In an in-vitro study of female rat trigeminal ganglia, estrogen-alfa receptors were localized in the cytoplasm of neurites and were associated with the upregulation of extracellular signal-regulated kinases (ERK-1), a protein associated with inflammatory pain and nociceptive neurons [27].

The pre-pubertal prevalence of migraine is 4%, equally in both boys and girls while post-pubertal prevalence is 18% for women and 6% for men, reflecting the prolific role of estrogen in the genesis of migraine [28]. The effects of the phase of the reproductive cycle on migraine genesis is outlined below (Table 3) [29].

**Table 3:** The association between the phase of the reproductive cycle and migraine

PHASE OF REPRODUCTIVE CYCLE	INCIDENCE (%)	COMMENT
Peri-menstrual	60	14% of migraines are exclusively menstrual
Pregnancy	23	De novo genesis of migraine during pregnancy is rare < 3%
Oral contraception	24.1	Develops within a month. Only 30-40 % improve with discontinuation
Menopause	14	No consistent response with estrogen replacement therapy or oophorectomy

In a study of 98 women undergoing luteinizing hormone therapy to suppress ovarian estrogen release, the fall in serum 17-beta estradiol correlated with a surge in migraine headaches in 84% of the women undergoing in-vitro fertilization [30].

Barometric pressure, atmospheric chemistry, and migraine

Meteoropathy is the study of pain generated by a change in barometric pressure and other parameters of weather systems such as humidity and temperatures. In a study of 34 patients with migraines who developed a headache while in hospital, migraine was found to occur when the atmospheric pressure lay between 1003 and 1007 hectoPascal (hPA), the standard pressure being 1013 hPA [31]. In a study of 28 migraine patients, a drop of barometric pressure greater than 5 hPA was associated with headaches in 14 patients. The barometric pressure was measured two days prior and after headache onset, with a rise in barometric pressure of greater than 5 hPA associated with a reduced frequency of headaches [32].

Rats exposed to low barometric pressure showed increased discharge in the spinal trigeminal nucleus in the receptive fields of the cornea but not in the temporal muscle or dura mater. Therefore, one would speculate that a drop in barometric pressure may lead more to orbital pain in migraineurs sensitive to barometric pressure drop [33]. In a rat chronic pain model, a drop in barometric pressure was associated with increased sympathetic nerve activity and an increase in pain induced by chronic constriction of the sciatic nerve. This effect is abolished by an inner ear lesion suggesting that the barometric pressure sensor influencing pain behavior may be located in the inner ear [34-35]. Cellular forskolin (c-fos) expression, a marker of neuronal activity, was increased in the superior vestibular nucleus in rats subjected to a 5-27 hPA drop in barometric pressure [36].

Thunderstorms and hot desert winds generate positive ions and increase the ratio of positive-to-negative ions in inhaled air, which increases blood and brain serotonin levels. Negative atmospheric ions, in particular, superoxide ions, oxidize serotonin into tryptamine-4,5-dione and reduce serum and brain serotonin. Superoxide ions are generated by two mechanisms, a salubrious effect with the Lenard effect and a potentially harmful one with corona lightning discharge (Table 4) [37].

**Table 4:** Cations and anions generated by natural phenomena and their biological effects SOD: superoxide dismutase

	LENARD EFFECT	LIGHTNING DISCHARGE
SOURCE	waterfalls, rain, cosmic rays, ultraviolet rays	lightning, corona discharge
ION SPECIES	superoxide only, attached to microclusters of water molecules	superoxide and ozone
HALF-LIFE OF ION SPECIES	60 seconds	few seconds
ACTIVITY ON SOD	increase	decrease

The Lenard effect is the shearing of water molecules by spray electrification and the generation of negative superoxide ions, an effect common in waterfalls. The Lenard effect may improve red blood cell elasticity and improve aerobic metabolism. Low concentrations of superoxide ions, generated by the Lenard effect, stimulate the activity of superoxide dismutase (SOD), an enzyme that accelerates the metabolism of harmful free radicals [38-39].

A controversial but intriguing phenomenon is the "serotonin irritation syndrome," a clinical phenotype that has overlapping but less severe symptoms than the serotonin syndrome and is thought to arise during thunderstorms and with hot and dry desert winds (Table 5) [40-41].

**Table 5:** Comparison between the serotonin syndrome and the serotonin irritation syndrome 5-HT: 5-hydroxytryptophan; 5-HIAA: 5-hydroxyindoleacetic Sferics are very low-frequency radio waves generated by lightning in the ionosphere.

	SEROTONIN SYNDROME	SEROTONIN IRRITATION SYNDROME
TRIGGER	Two or more serotonergic agents with different mechanisms of action: drug-induced - a toxic syndrome (toxidrome)	Atmospheric sferics - cation excess - likely serotonin release from the hypothalamus: atmospheric induced - thunderstorm, hot and dry desert wind
CLINICAL PHENOTYPE	Triad: encephalopathy, autonomic, motor hyperactivity	Triad: irritability, pain, and autonomic
TREATMENT	Serotonin antagonists - cyproheptadine, benzodiazepines, and dopamine antagonists	Anion ionizers
MECHANISM	Serotonin excess at synapses: 5HT-1A and 5-HT-2A	Cation-induced serotonin dysfunction: increased serum serotonin and reduced urinary metabolite (5-HIAA)

Alcoholic beverage and food-induced migraine headaches: pharmacology and biochemistry

One-third of migraine patients are sensitive to alcohol. Histamine is stored in the granules of mast cells (tissues) and circulating basophils. Centrally, it is found in high concentration in the hypothalamus. When injected experimentally into the hypothalamus, serotonin is released into the circulation. Histamine intolerance is due to diamine oxidase deficiency with impaired degradation of histamine. Red wine has 20-200 times the content of histamine than white wine. Histamine, an indicator of hygiene in foods, is released by food tissue mast cells. Alcohol can inhibit diamine oxidase leading to a higher histamine content of food. Red wine through two flavonoid extracts can lead to the release of serotonin into the blood from platelets. Meanwhile, white wine contains more sulfites than red wine. Sulfites can induce histamine release from mast cells [42], whereas a histamine H1 and H2 receptor blockade does not abort migraine headaches, the H3 receptor agonist has recently shown efficacy with aborting migraine headaches in small pilot clinical trials. The H3 and H4 receptors are found in the central nervous system (neurons and immune cells) and regulate neurogenic inflammation in the TVS. The H3 receptor is an auto-receptor and reduces the release of histamine [43].

Through the polyphenol resveratrol, both red and white wine can inhibit monoamine oxidase (MAO) activity and the reuptake of serotonin, thereby increasing its synaptic concentration. Red, more than white, wine can induce nitrous oxide release from endothelial cells, leading to direct vasodilatation or via CGRP release. Phenolsulfatransferase (PST), which is found in the highest concentration in the intestine, exists in two forms: PST-M, which inactivates phenolic monoamines (tyramine, dopamine, and norepinephrine), and PST-P, which inactivates phenol, p-cresol, and synthetic phenols. Both enzymes catalyze sulfate conjugation. Phenolic compounds are found in a wide variety of foods, including fruits, dairy products, and alcoholic beverages. Ethylacetate and the flavonoids, found more in red more than white wine, can inhibit PST-P, which with other alcoholic drinks also inhibit platelet PST-P. Patients with dietary migraines have lower levels of platelet PST-P and PST-M than non-dietary migraine-sensitive patients [44]. Ethanol also activates TRPV-1 in guinea pigs in trigeminal sensory afferents and activates TVS, thereby inducing neurogenic inflammation in meningeal blood vessels. This effect is blocked by CGRP antagonists [45].

A list of common migraine-provoking foods/beverages and their mechanisms of action are listed below. We add the pharmacological agent nitroglycerin to this list due to its ubiquity and historical significance (Table 6) [46].

**Table 6:** Migraine-provoking foods and agents and their mechanisms of action PEA: phenylethylamine; mg: milligram

SYNONYM	FOOD/BEVERAGE/AGENT	INCITING AGENT	MECHANISM	NOTES
Hot dog headache	hot dogs and cured meat	sodium nitrite	nitric oxide-induced vasodilation	
Chinese restaurant syndrome	soups and soy sauce	monosodium glutamate	glutamate-induced acetylcholine release	Headache, flushing, and palpitations: controversial syndrome.
Hangover headache	high congener beverages such as cognac and bourbon	aldehydes, methanol, and fusel alcohols (products of fermentation)	alcohol dehydrogenase induced lactic acidosis with altered redox state and accumulation of acetaldehyde	women have lower levels of alcohol dehydrogenase
Dynamite headache	nitroglycerin (dynamite factories), nitroglycerin sublingual (angina pectoris).	nitrous oxide	vasodilation	can be associated with amaurosis fugax
Chocolate headache	chocolate	PEA	PEA crosses the blood-brain barrier, increases brain serotonin and cerebral blood flow	As low as 3 mg PEA can induce a headache, blocked by methysergide

Stress, sleep disturbance, hypothalamus, and allostatic loading

The concept of allostatic load refers to the pathophysiology of cerebral neuronal networks by repetitive physiological and psychological stressors. The hypothalamus is a major component of the central autonomic network and the key nexus in homeostatic regulation. The hypothalamus activates the SSN-SPG-GSPN parasympathetic arc, which mediates parasympathetic vascular tone and the activation of perivascular nociceptive fibers and is connected with the TVS [47]. It is well-known that a sphenopalatine block can abort a migraine headache and cranial autonomic symptoms can occur in up to two-thirds of migraine patients ipsilateral to the headache. It is also well-established that PET scans show the activation of the hypothalamus in addition to the midbrain (PAG) and dorsal pons (LC and DRN) during a migraine headache attack [48].

The hypothesis is that stressors such as sleep disturbance, hunger, emotional distress, and even cyclical events (seasonal changes, menopause, menstrual cycle) activate the hypothalamus indirectly via the SSN-SPG-GSPN arc or directly activate the TVS. Activation of the TVS cascades into the amygdala and basal forebrain explaining the irritability, emotionality, and mental fog associated with migraine [49].

These repeated stressors (allosteric load) over the long term lead to reduced gray matter volume of the cingulate and insula (the salience network) altering the valence or value of incoming afferent signals thereby altering the perception of pain and altering the emotional response to pain (anxiety and depression). This phenomenon correlates with migraine frequency [50].

## Conclusions

Migraine triggers are quite common and highly variable. Identifying and eliminating migraine triggers improve the quality of life of migraineurs. From the scientific point of view, migraine triggers are fascinating, as they allow the neuroscientist a window into the function of the human brain and exposes the vulnerability of biological systems to nature. From the sferics of cations in the atmosphere altering brain serotonin turnover via barometric pressure-induced altered activity of the vestibular apparatus, to the docking of volatile odorants on the nasal mucosa setting off a cascade of events to the biochemical mechanisms of alcohol and the chemical constituent of foods to fluctuations of estrogen levels, ultimately the expression of migraine involves activation of the trigeminovascular system and the parasympathetic arc, SSN-SPG-SGPN. These downstream pathways are modulated by the dorsal pons, limbic system, hypothalamus, and the salience network, which explains many of the clinical manifestations of migraine.
